# Insights on the functional composition of specialist and generalist birds throughout continuous and fragmented forests

**DOI:** 10.1002/ece3.5204

**Published:** 2019-04-30

**Authors:** Luiz dos Anjos, Gabriela Menezes Bochio, Hugo Reis Medeiros, Bia de Arruda Almeida, Barbara Rocha Arakaki Lindsey, Larissa Corsini Calsavara, Milton Cezar Ribeiro, José Marcelo Domingues Torezan

**Affiliations:** ^1^ Departamento de Biologia Animal e Vegetal, Laboratório de Ornitologia e Bioacústica Universidade Estadual de Londrina Londrina, Paraná Brazil; ^2^ Programa de Pós‐Graduação em Ciências Biológicas Universidade Estadual de Londrina Londrina Paraná Brazil; ^3^ Departamento de Ecologia, Laboratório de Ecologia Espacial e Conservação – LEEC UNESP São Paulo Rio Claro Brazil; ^4^ Programa de Pós‐Graduação em Ecologia de Ecossistemas Aquáticos Continentais Universidade Estadual de Maringá Maringá Paraná Brazil; ^5^ Departamento de Biologia Animal e Vegetal, Laboratório de Biodiversidade e Restauração de Ecossistemas Universidade Estadual de Londrina Londrina Brazil

**Keywords:** beta diversity, forest fragments, functional diversity, passerines birds, southern Brazil

## Abstract

A decline in species number often occurs after forest fragmentation and habitat loss, which usually results in the loss of ecological functions and a reduction in functional diversity in the forest fragments. However, it is uncertain whether these lost ecological functions are consistently maintained throughout continuous forests, and so the importance of these functions in continuous forests remains unknown. Point counts were used to assess both the taxonomic and functional diversity of specialist and generalist birds from sampling in a continuous primary forest compared with forest fragments in order to investigate the responses of these groups to forest fragmentation. We also measured alpha and beta diversity. The responses of specialists and generalists were similar when we assessed all bird species but were different when only passerines were considered. When examining passerines we found lower total taxonomic beta diversity for specialists than for generalists in the continuous forest, while taxonomic beta diversity was higher in the fragmented forest and similar between bird groups. However, total functional beta‐diversity values indicated clearly higher trait regularity in continuous forest for specialists and higher trait regularity in fragments for generalists. Specialists showed significantly higher functional alpha diversity in comparison with generalists in the continuous forest, while both groups showed similar values in fragments. In passerines, species richness and alpha functional diversity of both specialist and generalist were explained by forest connectivity; but, only fragment size explained those parameters for specialist passerines. We suggest that considering subsets of the community with high similarity among species, as passerines, provides a better tool for understanding responses to forest fragmentation. Due to the regularity of specialists in continuous forest, their lost could highly affect functionality in forest fragments.

## 1 INTRODUCTION

Deforestation caused by anthropogenic activities has a significant impact on biodiversity and ecological processes (Haddad et al., 2015). Species exhibit different levels of sensitivity to forest loss and fragmentation, which makes some of them more prone to local extinction in a given forest fragment, while others persist during different temporal lags (Tilman, May, Lehman, & Nowak, 1994; Fahrig, 2003; Metzger et al., 2009; Laurance et al., 2011). Habitat fragmentation may reduce population of some species by disrupting connectivity between habitats. However, matrix permeability may counteract the negative effects of fragmentation by increasing both functional connectivity among habitat patches and rescue effects that contribute to recolonization of forest fragments by other species (Laurance et al., 2011). Generalist species may be particularly favored during this process of recolonization, due to plasticity in food and/or habitat use, as documented for amphibians, birds, and mammals (Newbold et al., 2015), which drives changes in species composition in forest fragments (De Coster, Banks‐Leite, & Metzger, 2015). Habitat loss and fragmentation reduce vegetation heterogeneity resulting in the loss of filters of pristine forest (Newbold et al., 2015; De Coster et al., 2015; Morante‐Filho, Arroyo‐Rodríguez, & Faria, 2016). Sensitive species frequently exhibit some kind of specialization (e.g., restrict diet and habitat) that makes them more prone to extinction in forest fragments (see Henle, Davies, Kleyer, Margules, & Settele, 2004).

Forest loss and fragmentation can result in decrease in the number of sensitive species and changes in species composition, which impacts functional diversity, however different groups of organisms may show distinct responses (see Flynn et al., 2009). In birds, a decline in species number after deforestation usually causes the loss of ecological functions and drives down functional diversity in forest fragments (Sekercioglu et al., 2002). However, it is uncertain whether functional diversity lost in a fragmented landscape is consistently maintained throughout a continuous forest prior to habitat loss and fragmentation. Studies have indicated that continuous forests, in both temperate and tropical zones, are not uniform but patchy environments (Holmes, 1990; Karr, 1990). If sensitive species are poorly represented in patches throughout the continuous forest, their impact on the overall functional diversity of the community after fragmentation should be low. In contrast, if these species are regularly found along large tracts of forest, the loss of sensitive species should have a substantial impact on ecosystem functions.

In previous studies, we have shown that the area of forest fragments positively influences bird species richness in a fragmented landscape of northern Paraná in southern Brazil, with more accentuated influence on sensitive species (Anjos, 2006; Anjos, Bochio, Campos, McCrate, & Palomino, 2009; Medeiros, Bochio, Ribeiro, Torezan, & Anjos, 2015). Here, we used point count survey data to investigate the diversity of generalist and specialist bird species and shifts in their traits between primary continuous forest and fragmented landscape, in the same region. We aimed to investigate how taxonomic and functional diversity of birds respond to habitat composition and configuration in continuous and fragmented Atlantic forest landscapes in southern Brazil. We assessed alpha and beta diversity of generalist and specialist bird species to detect shifts in their traits between a primary continuous forest and a fragmented landscape. Beta diversity represents the dissimilarity among communities and allows us to investigate different hypotheses to describe the process that drives species distribution: nestedness and spatial turnover (Baselga, 2010). Indeed, beta diversity is an important tool for biodiversity conservation (Whittaker, 1960; Clough et al., 2007) and can be used to identify sites of particular importance for the maintenance of regional diversity (Davidar, Yoganand, & Ganesh, 2001), even sites that could temporally control the source–sink dynamic (Ruhí, Datry, & Sabo, 2017). Such approach has intensified the recent debate on the relative importance of habitat amount and fragmentation for explaining biodiversity patterns (see Fletcher et al., 2018; Fahrig et al., 2019).

Studies in the Brazilian Atlantic forest have found that taxonomic beta diversity of birds tends to be higher in specialist species than in generalist species with a reduction in forest cover (Morante‐Filho et al., 2016). Therefore, we expect that taxonomic diversity and functional diversity of specialists and generalists differ in continuous and fragmented landscapes.

We addressed the following questions for specialist and generalist bird groups: (a) Do spatial distribution and functional diversity differ between bird groups throughout the continuous forest? (b) Do the bird groups respond differently to forest fragmentation? and (c) Are species and functional diversity in both groups influenced more by local vegetation integrity or by landscape functional connectivity? Our expectations are as follows: (a) Specialist species are more patchily distributed along the continuous forest block because species are more likely to be attached to microhabitats (e.g., Stratford & Stouffer, 2013; Powell et al., 2015), which results in higher taxonomic and functional beta diversity than for generalists; (b) taxonomic and functional beta diversity of specialists is higher in fragmented forest than in continuous forest while the beta diversity of generalists is not affected by fragmentation. This is expected because specialist birds are more sensitive to forest fragmentation (Morante‐Filho et al., 2016) than generalists (Newbold et al., 2015); and (c) the specialists are more affected by local vegetation integrity, forest amount, and forest connectivity than the generalists in fragmented landscapes (see Laurance et al., 2011).

## 2 METHODS

### 2.1 Study area

**Figure 1:**
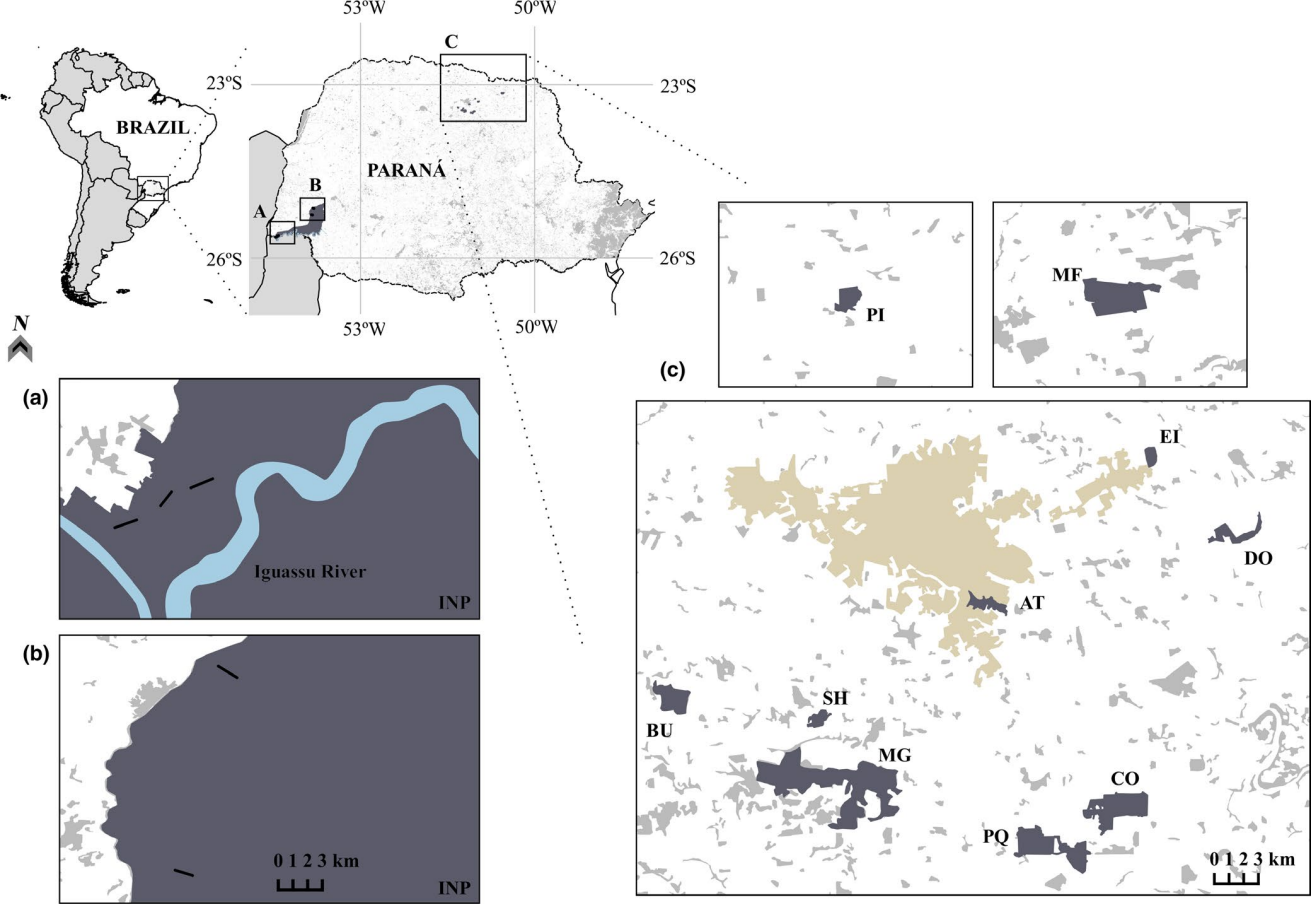
The Iguassu National Park (a and b) and the fragmented forest landscape (c); black lines represent the five transects in the Iguassu National Park (INP) and letters (such as PI and MF) are the codes of the studied forest fragments (in dark gray). In brown are the cities of Londrina and Ibiporã, northern Paraná State, southern Brazil

Two regions were sampled including a continuous forest and forest patches within a highly fragmented landscape. Five 1‐km transects were sampled in a large block of continuous forest in the Iguassu National Park (INP), which is located in southwestern Paraná State in southern Brazil and covers an area of approximately 187,000 ha (Figure 1). Ten forest fragments (60–866 ha in size) were evaluated in a fragmented landscape localized in northern Paraná State 300 km from the INP (Figure 1). The forest fragments are located in landscape contexts with varying degrees of functional connectivity and forest cover (see section 2.7 Landscape metrics and data analysis).

The native vegetation of both continuous and fragmented forest landscapes is seasonal semideciduous forest (SF). Compared with Brazilian Atlantic coast rainforests, SF vegetation has a taller and fewer dense canopy, taller emergent trees, denser understory, less vascular epiphytes, and more woody lianas (IBGE, 2012). There is a weak dry season extending from June to August with a monthly rainfall of <50 mm. The annual average temperature ranges from 19 to 22°C, and annual rainfall ranges from 1,400 to 1,600 mm. In all sampling sites, the soil is a deep, fertile, and well‐drained eutroferric red nitrosol, with a smooth terrain that is almost devoid of steep surfaces (Maack, 1981). Due to deforestation, only 7% of the SF remains, and it is represented mainly by small and isolated forest fragments (Ribeiro, Metzger, Martensen, Ponzoni, & Hirota, 2009).

### 2.2 Bird groups

Bird species were considered specialists or generalists based on their diet. We used the database of Wilman et al. (2014) and considered species that feed on single item (e.g., invertebrates, vertebrates, fruits, nectar, seeds, or other specific plant materials) at a percentage ≥ 70% as specialists. All other species were considered generalists. We analyzed data for all birds and for passerines separately. We used the taxonomy of the South American Classification Committee of the American Ornithological Society (SACC; Remsen et al., 2018) to separate the passerines from the other birds.

### 2.3 Field surveys

Bird surveys were conducted in 15 forest sample units that encompass five sites of continuous forest within the INP and 10 forest fragments in the fragmented landscape. In each forest sample unit, we fixed a 1‐km transect composed of six point counts which were 200 m away from each other. The three transects of the western INP region were separated from each other by a minimum of 1 km and are located at an altitude of 180–270 m. The other two transects are located in the eastern portion of the INP, at a higher altitude (between 470 and 680 m; Table 1). The five transects were selected to capture variations in bird communities along the INP. In total, 30 points were sampled in the INP while 60 points were performed in the forest fragments (1‐km transect in each fragment). All transects were carried out in the forest interior, at least 300 m from the forest border. Every morning, six points of one transect were sampled in a sequence (according to Blondel, Ferry, & Frochot, 1970; Bibby, Burguess, & Hill, 1993). Beginning at sunrise, the observer sampled each point for 15 min, with a 15‐min break between the points. Four samplings were obtained for each transect. The detection radius at each point was 50 m. Although point counts allow us to calculate the relative abundance, only the lists of species for each transect were used in the present study. The three transects of the western part were sampled once in the spring of each year from 2011 to 2014, while the other two transects of the eastern portion of the INP were sampled in the spring and summer of 2004 and 2005. Although sampling was performed in different years, no anthropogenic action occurred in the transects during this period as the INP is a protected reserve. Surveys in the forest fragments were performed four times per fragment in the spring and summer of 2010 and 2011. Forest fragments and their transects were also not affected by anthropogenic actions during this period.

**Table 1:** Coordinates of transects, where point counts were sampled for bird surveys, in the Iguassu National Park (INP) and in forest fragments (size) in the north of Paraná

Transects (region in INP)	Coordinates
(1) Western region	25°37′34.8′′S/54°27′35.6′W
(2) Western region	25°36′53.3′′S/54°26′08.3′′W
(3) Western region	25°36′16.5′′S/54°25′01.6′′W
(4) Eastern region	25°07′ 54′′S/53°48′40′′W
(5) Eastern region	25°14′ 35′′S/53°50′12′′W
**Forest fragments (size)**	
MG (650 ha)	23°26′54.28′′S/51°14′42.00′′W
MF (876 ha)	23°09′37′′S/50°34′00′′W
AT (85 ha)	23°20′41′′S/51°48′23′′W
EI (60 ha)	23°15′21′′S/51°01′53′′W
PI (74 ha)	22°46′49′′S/51°29′21′′W
PQ (542 ha)	23°30′05′′S/51°04′39′′W
CO (564 ha)	23°28′12′′S/51°02′50′′W
BU (288 ha)	23°24′19′′S/51°19′31′′W
DO (166 ha)	23°18′05′′S/50°59′11′′W
SH (85 ha)	23°24′38′′S/51°14′09′′W

Vegetation integrity in both continuous forest and forest fragments was evaluated through the rapid ecological assessment method (REA), which is based on variables of SF plant community structure, such as the presence of endangered species and exotic species, and density of standing dead trees and vine tangles. For a full description of the REA method and variables, see Medeiros and Torezan (2013). Evaluations by REA were conducted in the same transects used for the bird surveys.

### 2.4 Species traits

We used two trait data sets. The first data set was used for all birds and concerned four functional traits: diet, foraging forest strata, body mass (all based on the data set of Wilman et al., 2014), and morphological measurements. The morphological measurements were beak length, beak height, beak width, tail length, and wing length, obtained in the Zoology Museum of São Paulo (MZUSP); details on how measurement procedures were carried out are located in Appendix A1. All trait data are available in Data S1.

The second set of traits was only recorded for passerines, for which we used five functional traits: foraging substrate, foraging orientation, locomotion strategy, body mass, and morphological measurements. Those traits were selected due to the large radiation of passerines in terms of foraging techniques. Details on this second set of traits and references for relevant sources of information are found in Appendix A2 and Data S2.

### 2.5 Beta diversity

To test whether the species and traits of the bird groups differ in their spatial distribution between continuous and fragmented forests, we calculated a measure of beta diversity and partitioned it into nestedness and turnover (Baselga, 2010). Nestedness of species assemblages occurs when the biota of sites with lower species richness are subsets of the biota of richer sites (Almeida‐Neto, Guimaraes, Guimarães, Loyola, & Ulrich, 2008). In contrast, spatial turnover implies the replacement of some species by others as a consequence of environmental sorting or spatial and historical constraints (Baselga, 2010). We partitioned beta diversity into pure nestedness and spatial turnover, as proposed by Baselga (2010), for both taxonomic and functional beta diversity, as proposed by Villéger, Grenouillet, and Brosse (2013). Total beta diversity and its components were calculated for each bird group in each landscape. All calculations were carried out using the *betapart* package (Baselga & Orme, 2012) in R version 3.3.2 software (R Core Team, 2016). See Appendix A3 for details on beta‐diversity calculations.

### 2.6 Alpha diversity

Species richness and a functional alpha diversity index were used as response variables to investigate how the patch size, vegetation integrity, forest amount, and forest connectivity influence taxonomic and functional alpha diversity in both bird groups. There are various ways to calculate functional diversity; we used the functional dispersion metric (FDis; Laliberté & Legendre, 2010). FDis reflects the contrast between species in trait space, measuring both functional richness and divergence by the same index (Laliberté & Legendre, 2010). Therefore, FDis should increase when niche complementarities increase, due to abundance and species occurrence probabilities (Mason, Bello, Mouillot, Pavoine, & Dray, 2013). It can be used for both abundance and presence–absence data, and its calculation is not affected by species richness (Laliberté & Legendre, 2010). As we have traits represented by different numbers of trait characters, with continuous traits represented by only one character and categorical traits with a variable number of characters, different weights were assigned to each trait so that all traits had equal influence in the multivariate trait space. Thus, all traits were assigned proportional values for the calculation of the functional diversity indices by calculating the weight: Wi = 1/Ni, where Ni is the number of characters by which a categorical trait is divided into (Laliberté & Legendre, 2010). To compute FDis, the same species occurrence matrix that was used for beta‐diversity calculations, in addition to the traits per species matrix, was used. The functional distances were also computed between pairs of species according to the trait values by using the Gower distance (Gower, 1966). A PCoA was calculated from the matrix; the axes of the PCoA were used as “new” independent functional traits to generate a multivariate trait space. FDis was computed with the “dbFD” function of the package FD (Laliberté, Legendre, & Shipley, 2014) using R version 3.3.2 software (R Core Team, 2016). To test for differences in species richness and FDis between groups for each landscape, and between landscapes for each group, we used Welch two‐sample *t* tests, which accounted for differences in variances between factors.

### 2.7 Landscape metrics and data analysis

A binary map (forest vs. matrix) obtained from SOS Mata Atlântica and Instituto Nacional de Pesquisas Espaciais (2008), was used to calculate forest cover (%) and forest connectivity at four spatial scales (250, 500, 1,000, and 1,500 m) around each of the forest fragments. Forest connectivity is based on the summation of the patch size (ha) of all patches within a specified search radius surrounding the focal patch, which is weighted by the inverse of the distance between the edge of the focal patch and the edge of each of the other patches (Gustafson & Parker, 1992). Landscape metrics were calculated using ArcGIS (ERSI—Environmental Systems Research Institute, 2005) and the V‐LATE extension (LARG—Landscape & Resource Management Research Group, 2005).

To estimate the relative contribution of patch size, forest cover, forest connectivity, and vegetation integrity (REA) to explain bird diversity patterns, beta regressions were used for functional diversity indices and generalized linear models (GLM) with Poisson error structure were used for species richness. Each of the above explanatory variables was used to build four independent models. A null model, which represents the absence of effect, was also included in the set of competing models. To identify the best models, we used the Akaike information criterion (AIC; Burnham & Anderson, 1998) with the small sample correction (AICc; Hurvich & Tsai, 1989) in addition to the AICc weight (wAICc; Burnham & Anderson, 2002) and the AICc delta value (ΔAICc). The models with wAICc ≥ 0.10, ΔAICc ≤ 2.0, and *p*‐values ≤0.05 (model fit) were considered equally plausible in terms of explaining the dependent variables. All analyses were performed using R version 3.3.2 software (R Core Team, 2016). We used the packages “*betareg*” (Cribari‐Neto & Zeileis, 2010) and “*stats*” to fit beta regression and generalized linear models, respectively.

## 3 RESULTS

Overall, 161 bird species were recorded, comprised of 93 specialists and 68 generalists. A total of 127 species were recorded in the INP continuous forest; 72 species (57%) were specialists and 55 species (43%) were generalists, while 114 species were recorded within forest fragments, where 66 species (58%) were specialists and 48 species (42%) were generalists. Among the passerines, 106 species were recorded in total, in which 66 species were specialists and 40 species were generalists. In the INP, 81 passerines were recorded, including 50 specialists (62%) and 31 generalists (38%) while 75 passerines were recorded in the fragmented forest, with 46 specialists (61%) and 29 generalists (39%).

### 3.1 Bird diversity

**Figure 2:**
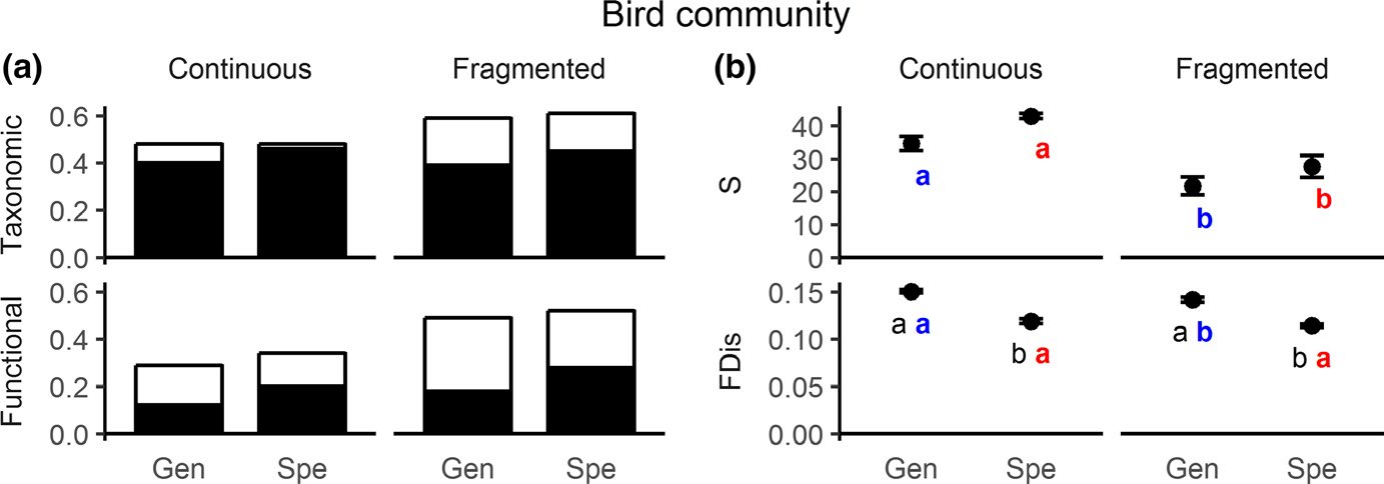
(a) Taxonomic and functional beta diversity and their components for specialists (spe) and generalists (gen) in continuous and fragmented forests for bird community. Bars represent total beta diversity. Black portions of the bars represent the contribution of the turnover component, and white portions represent the contribution of the nestedness component. (b) Mean and standard error bars for species richness (s) and functional diversity (FDis) for specialists and generalists in continuous and fragmented forest sites for bird community

We did not find differences in total taxonomic beta diversity between generalist and specialist birds in continuous and fragmented forest. Total functional beta‐diversity values were slightly lower for generalist species than for specialists in both continuous and fragmented forests (Figure 2a). Overall, total beta diversity was lower for both generalists and specialists in the continuous forest than in the fragments, especially for functional beta diversity. In general, the most important component for taxonomic beta diversity was turnover (Figure 2a). Values of taxonomic turnover were similar between continuous forest and fragments, while the importance of nestedness was higher for fragments than continuous forest. On the other hand, functional beta diversity showed an overall higher contribution of nestedness, but with slightly higher turnover for specialists in both forest types (Figure 2a). Overall, we found that nestedness made a small contribution to changes in taxonomic beta diversity, but made a higher contribution to functional beta diversity, indicating species substitution between sites, but low trait substitution.

Specialists showed significantly lower FDis in comparison with generalists in both continuous and fragmented forest (Figure 2b). Generalists and specialists showed lower taxonomic richness in fragments than in the continuous forest. However, only generalists showed lower FDis in fragments. Thus, while generalists showed reductions in both number of species and contributions to ecological functions with fragmentation, the reduction in species number did not seem to have affected the functional contribution of specialists. Species richness of specialists was positively related to forest connectivity and fragment size, while generalist species was only positively related to forest connectivity. On the other hand, FDis showed significant relationships with landscape predictors only for generalists, being positively related to REA and fragment size (Table 2; Figure S1).

**Table 2:** Plausible models to explain the species richness (S) and functional diversity (FDis) of specialist and generalist species within both the entire community and passerine group in continuous forest and fragmented landscapes in Paraná State, southern Brazil

Dependent variable		Model	∆AICc	wAICc
Total community	S (specialists)	~Proxy	0	0.65***
		~Size	1.7	0.29***
	FDis (specialists)	~Null	‐	‐
	S (generalists)	~Proxy	0	0.79***
	FDis (generalists)	~REA	0	0.63***
		~Size	1.8	0.25***
Passerines	S (specialists)	~Proxy	0	0.52***
		~Size	0.4	0.42***
	FDis (specialists)	~Null	‐	‐
	S (generalists)	~Proxy	0	0.73**
	FDis (generalists)	~ Null	‐	‐

Note

Asterisks indicate the level of significance of the models (model fit). Explanatory variables: forest connectivity at 1,000 meters radius (Proxy), fragment size (Size), and vegetation integrity (REA).

Abbreviations: ∆AICc: delta value of AICc; AICc: Akaike information criterion with the small sample correction; and wAICc: weight of evidence of the models.

** *p* < 0.01.

*** *p* < 0.001.

### 3.2 Passerine diversity

**Figure 3:**
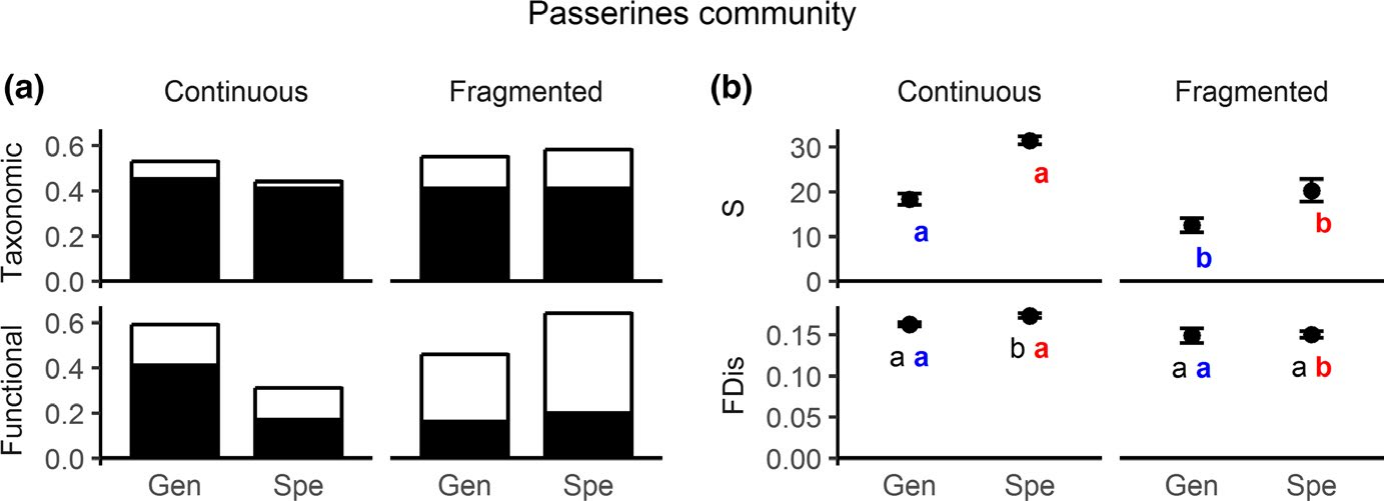
(a) Taxonomic and functional beta diversity and their components for specialists (spe) and generalists (gen) in continuous and fragmented forests for passerines community. Bars represent total beta diversity. Black portions of the bars represent the contribution of the turnover component, and white portions represent the contribution of the nestedness component. (b) Mean and standard error bars for species richness (s) and functional diversity (FDis) for specialists and generalists in continuous and fragmented forest sites for passerines community

For passerines, we found lower total taxonomic beta diversity for specialists than for generalists in the continuous forest. In the fragments, taxonomic beta diversity was higher and similar between bird groups (Figure 3a). On the other hand, total functional beta‐diversity values showed marked differences between bird groups, with lower functional beta diversity observed for specialists than for generalists in the continuous forest; the opposite pattern was observed between bird groups in the forest fragments (Figure 3a). Similar to when we considered the entire bird community, the most important component for passerine taxonomic beta diversity was turnover (Figures 2a and 3a). Values of taxonomic turnover were similar between continuous forest and fragments, with nestedness being of low importance. Conversely, functional beta diversity showed an overall higher contribution of nestedness, especially in the fragments. We found a marked difference in specialist trait nestedness between continuous and fragmented forest, which led to an increase in total functional beta diversity for this group in the forest fragments (Figure 3a). This suggests that for passerines, there is a more clear difference in functional trait regularity between specialists and generalists, with higher trait regularity in continuous forest for specialists and higher trait regularity in fragments for generalists.

Specialists showed significantly higher FDis in comparison with generalists in the continuous forest, while both groups showed similar values in the fragments (Figure 3b). When comparing the mean values separately for each group between landscapes, a decrease in richness in forest fragments was observed for both bird groups (Figure 3b). FDis decreased for specialists moving from continuous to fragmented forest, but showed no significant difference for generalists. Species richness and FDis of specialists were affected by forest connectivity and fragment size, while generalists were only explained by forest connectivity (Table 2; Figure S2). This shows that more landscape predictors affected species and traits for specialists than generalists with forest loss and fragmentation.

## 4 DISCUSSION

Simultaneously analyzing taxonomic and functional alpha and beta diversities allowed us to interpret the responses of generalist and specialist birds in continuous and fragmented forests in relation to (a) variation in species richness and in functional dispersion and (b) dissimilarity in the composition of species and traits throughout forest types (see Si, Baselga, Leprieur, Song, & Ding, 2016). Specifically, this approach provides important insights on whether variation in species richness reflects a reduction in the diversity of traits that could affect ecosystem functionality (Díaz & Cabido, 2001; Cadotte, Carscadden, & Mirotchnick, 2011). We found distinct patterns for generalists and specialists when analyzing both the entire bird community and the community subset composed with passerines. Thus, we highlight that the interpretation of specialist and generalist bird responses to habitat loss and fragmentation depends on the group of birds analyzed and on the aspect of diversity evaluated (taxonomic or functional).

### 4.1 Bird diversity

We predicted that specialists (a) would have higher taxonomic and functional beta diversity than generalists in continuous forest, (b) but that the values of those metrics would decline in the forest fragments in this group, and (c) that they would be more affected by vegetation integrity and landscape parameters. Our results did not support our predictions that specialists have more patchier distribution than generalists, neither that continuous forest support higher functional diversity than fragmented landscape. Moreover, the beta diversity of specialists and generalists respond similarly to fragmentation. Only for FDis, we found different patterns for specialists and generalists as it was lower in fragments than continuous forest for generalists, but not for specialists. Finally, analyses using the total bird community revealed that generalists were more affected by vegetation integrity and landscape functional connectivity than specialists. Therefore, most of the predictions for how generalists and specialists species will react to forest fragmentation (see Laurance, 2010) were not confirmed in this study.

When we analyze all species of the bird community together, we compare birds with quite diverse ecological adaptations, which may mask distinct responses from different species groups. For example, the range in body masses and morphological measurements was considerable when considered, for example, tinamous, raptors, doves, hummingbirds, woodpeckers, and antbirds. Indeed, these species occupy very different ecological niches, as they have different adaptations and thus use the habitat in distinct ways, for example, in terms of including a large variety of food items (see Data S1). As a group passerines have smaller range of body masses and morphological measurements when considering, for example, antbirds, flycatchers, and tanagers. In our study, 66 species (out of 161 species total) were specialist passerines and among these, 58 species were insect specialists (see Data S2). We suggest that considering subsets of the community with high similarity among species provides a better tool for understanding responses to forest fragmentation.

### 4.2 Passerines diversity

According to our results, generalists showed a patchier distribution of traits than specialists in the continuous forests. At the same time, FDis was higher for specialists than for generalists in the continuous forest. We suggest that the low functional beta diversity and high FDis found in our study for specialist passerines in the continuous forest are possibly due to the smaller niche breadth in this group. Importantly, the high taxonomic turnover found for the specialists was not reflected in terms of high functional beta diversity in our study. This suggests a turnover of species with redundant traits. According to our results, it seems that the group of generalist passerines has more distinct traits when compared to specialists.

Passerines, with small body masses, should survive with less energetic requirements than nonpasserines. As they consume smaller food items, it is possible that they are better able to discriminate fine‐scale habitat variation (Jetz, Carbone, Fulford, & Brown, 2004; Reif, Hořák, Krištín, Kopsová, & Devictor, 2016). In fact, a study on the diversity of passerines in an elevation gradient showed that high species richness is mostly associated with denser occupation of the trait space (Pigot, Trisos, & Tobias, 2016). Niche packing in passerines was the most common tendency in the richest sites of the studied gradient (Pigot et al., 2016). The relationship between species richness and FDis, and the low values of beta diversity, indicate functional redundancy, suggesting the existence of niche packing in specialist passerines (see Pigot et al., 2016). In a worldwide analysis, Belmaker, Sekercioglu, and Jetz (2012) showed that diet specialization and species richness are strongly related. They found that this relationship is particularly strong in South America and is primarily due to high adaptive radiation in the passerine families Furnariidae and Tyrannidae, which have relatively narrow dietary niches. The majority of specialist passerines in the continuous forest of our study area belong to those families.

Functional beta diversity increased substantially for specialists in fragments, while decreasing for generalists, showing that specialist species are more sensitive to fragmentation. The difference in FDis between fragments and continuous forest observed for specialists gives further support to this hypothesis. Conversely, FDis of generalists showed no difference between fragmented and continuous forests.

Due to different functional tendencies between bird groups, it is possible that the ecological functions of specialists could be partially performed by generalists in the fragmented forest, as suggested in some studies (e.g., Newbold et al., 2015; De Coster et al., 2015). This is possible because generalists also eat food items consumed by specialists (see Data S2). Since the functions of specialists and generalists are unlikely to be exactly the same, the replacement of traits is incomplete and may not maintain functional integrity in the fragmented forest (De Coster et al., 2015). The low beta functional diversity found in the INP suggests that the functional traits of specialists are important in the continuous forest since their composition does not vary much through sampled sites. In our study, 43 insect specialist passerine species were recorded from the continuous forest, which represents 34% of the total birds recorded in the INP. The loss of insectivorous birds due to forest fragmentation, particularly from the understory, has been frequently documented in the literature (see Stratford & Stouffer, 2013; Powell et al., 2015). According to our results for continuous forest, the loss of those species should have an important impact on the functionality of fragmented forests.

The high importance of nestedness for specialists in the fragmented forest means a loss of trait combinations among fragments, indicating that some communities may represent subsets of others. Because both species richness and FDis were positively related to forest connectivity and fragment size in specialists, a result commonly found in the literature (see Laurance et al., 2011), it is possible to suggest that some subsets of species and traits in this group are restricted to larger, nonisolated forest fragments. On the other hand, although functional beta diversity of generalist species was reduced in fragments, this reduction was linked to a reduction in turnover, while nestedness actually increased. Generalists also had similar losses of species as did specialists, but generalist FDis was similar in both landscapes. This suggests that FDis of specialists is more affected by forest loss and fragmentation than generalist species.

### 4.3 Beta diversity and implication for conservation

Studies on fragmentation have highlighted the importance of measuring beta diversity in fragmented landscapes (see Fahrig, 2003; Tscharntke et al., 2012; Fahrig et al., 2019). It has been indicated that a set of several small forest fragments support higher number of species, even of specialist and threatened species, than few but large fragments suggesting that the spatial arrangement of habitats (landscape configuration) matters (see Fahrig, 2017; Fahrig et al., 2019). Those results would reflect the habitat amount effect and have highlighted the importance of small forest fragments (see Fahrig et al., 2019). Aside from bringing an unexpected result, the revision of Fahrig (2017) suggests a higher importance of small forest fragments than has been previously believed (see Fletcher et al., 2018) Although we did not compared two different sets of forest fragments, some interpretations are possible, in particular on ecosystem functionality. In our study, taxonomic beta diversity was similar or slightly higher in fragments than in the continuous forest for both generalists and specialists, but species richness was always lower in fragments than in the continuous forest (see Figures 2 and 3). Habitat amount hypothesis predicts that species richness increases with increasing habitat amount regardless of landscape configuration (Fahrig, 2003; Fahrig et al., 2019). In contrast, we found that landscape configuration (forest connectivity) affected both specialist and generalist bird species, as suggested frequently by studies on forest fragmentation (see Fletcher et al., 2018). Moreover, habitat amount was important at local scale (fragment size), but forest cover at landscape scale did not explain the patterns. In addition, there was high species turnover but low traits substitution in fragments for the overall bird community. We do not know how crucial the loss of trait heterogeneity is for the long‐term functionality of forest fragments, in particular for the highly sensitive specialist passerines (see Figure 3). In our study, specialist passerines were clearly more sensitive than generalist passerines. Therefore, we suggest that a broader view of beta diversity, which includes functionality, would improve accuracy in the evaluation of the importance of small forest fragments.

We used only incidence data to calculate all our diversity metrics, as this was the only available information for all sites. It is then important to remember that all our indexes account only for composition, including FDis. In this case, FDis represents the occupation of the functional space by the species present in the communities and provides no information on the distribution of individuals on the functional space (Laliberté & Legendre, 2010). A further development of the approach we have used in this study is to have data on abundance of bird species, which would add different information to our analysis, and could bring different results, mainly considering the functional structure of the community, which could be better explored with the use of other functional diversity indexes.

## 5 FINAL CONSIDERATIONS

The results did not confirm our predictions on differences between specialists and generalists when all bird species were considered. Therefore, our study of passerines indicates that the evaluation of a given subgroup of the community may reveal biological responses to forest fragmentation that are otherwise unseen. Furthermore, our results indicate an important role of specialist passerines due to their regularity in trait composition in the continuous forest, which is lost with decreasing fragment size and connectivity in the fragmented forest. Thus, we highlight that the protection of large and well‐connected forest fragments is very important in fragmented landscapes, in order to maintain functional diversity and community composition of specialist passerines.

## AUTHOR CONTRIBUTIONS

L.d. Anjos conceived the idea of the study and collected data in the Iguassu National Park. G. M. Bochio collected data in the forest fragments of northern Paraná State. G. M. Bochio, B. A. Almeida B. R. A. Lindsey, and L. C. Calsavara measured the bird specimens in the Zoology Museum of São Paulo and carried out the analyses related to functional alpha and beta diversity. H.R. Medeiros, M. C. Ribeiro, and J.M.D. Torezan worked with the landscape metrics and models in the fragmented landscape. All authors contributed to the text after the first draft was written by L.d. Anjos. All authors gave final approval for publication.

## DATA AVAILABILITY

The dataset supporting this article are available in the Dryad Repository https://doi.org/10.5061/dryad.r69v7gp.
