# Supplementary material for: Insights on the functional composition of specialist and generalist birds throughout continuous and fragmented forests
Source: Ecol Evol. 2019 Apr 30;9(11):6318–28. doi: 10.1002/ece3.5204 (PMC6580428; doi:10.1002/ece3.5204)
Supplement: Supplementary file 1 [file ECE3-9-6318-s001.doc]

**Supporting Information**

**A1.** Information on morphological attributes: wing and tail length and beak (length, height and width) were measured for five specimens of each species in the Museum of Zoology of the University of São Paulo (MZUSP). In order to obtain these morphological measurements, we used a caliper rule to obtain measurements of beak length (from opening to tip of the beak), beak height (vertical width at the nostrils) and beak width (horizontal width at the nostril), wing length (from insertion of primary feathers to wingtip), and tail length (insertion of rectrices to tail tip).

**A2.** Information on functional traits used for passerines: importance values were assigned to the functional traits for specialists and generalists groups, and all trait information was obtained from the literature: Krabbe and Schulenberg (2003a, b), Remsen (2003), Whitney (2003), Zimmer and Isler (2003), Chesser (2004), Snow (2004a,b) and Fitzpatrick et al. (2004). For each trait character, we assigned the value 3 when the literature above suggests that species gives importance for that character, value 2 when the importance is not mentioned, only suggesting that species exhibits that character, value 1 when the literature mentioned that species only eventually exhibits the character, and value 0 for absence.

**A3.** Information on the procedure to evaluate Beta diversity: for both species and trait compositions, three beta diversity metrics were calculated: a) Sorensen dissimilarity index (β_sor_), representing total variation between assemblages, which incorporates both turnover and nestedness components; b) Simpson dissimilarity index (β_sim_), measuring differences between assemblages due to species turnover; and c) β_sne_, which results from the difference between β_sor_ and β_sim_ and represents the dissimilarity due to nestedness. This was done separately for specialists and generalists, and for continuous and fragmented forests, so that we have two taxonomic and two functional beta diversity metrics for both continuous forest and fragmented forests. For taxonomic beta diversity, we used a species occurrence matrix per bird group for each forest landscape. A dissimilarity matrix of Sorensen was calculated from each species occurrence matrix, where we compared species dissimilarity between all sites in the landscape. The multiple-site dissimilarity metric accounts for different degrees of overlap from species shared by more than two sites (Baselga, 2010). For functional beta diversity, we used the same species occurrence matrix used for taxonomic beta diversity plus the traits per species matrix. We computed the functional distances between pairs of species according to the traits’ values, using the Gower distance. The Gower distance is appropriate for dealing with a matrix of mixed traits (both continuous and categorical; Gower, 1966). From this matrix, we summarized the trait data using a principal coordinates analysis (PCoA). The three first axes of the PCoA were used as “new” independent functional traits to generate a multivariate trait space. A convex hull was then projected onto this space for each bird assemblage, with vertices of the hull defined by the species trait values (Villéger et al., 2008). Functional beta diversity calculations are based on the volume of convex hull intersections in this multidimensional functional space (Villéger et al., 2013). We compared the results of the beta diversity partitioning using proportions in which the value of the total beta diversity (β_sor_) represents 100% and the turnover and nestedness components are proportional to it. Comparisons between multiple-site beta diversity measures are influenced by the number of sites and thus should be always conducted among areas with equal numbers (Baselga, 2010). Therefore, in order to make beta diversities computed for sets with different number of sites comparable (5 for continuous and 10 for fragmented areas), beta values for the fragmented forest were computed using a resampling procedure, taking 100 random samples of five inventories and then computing the average for beta diversity and its components.

**Figure S1.** Best supported models of the relation between the explanatory variables and species richness (S) and functional diversity (FDis) of specialists and generalists species within bird community in continuous and fragmented forest landscapes in southeast Brazil.


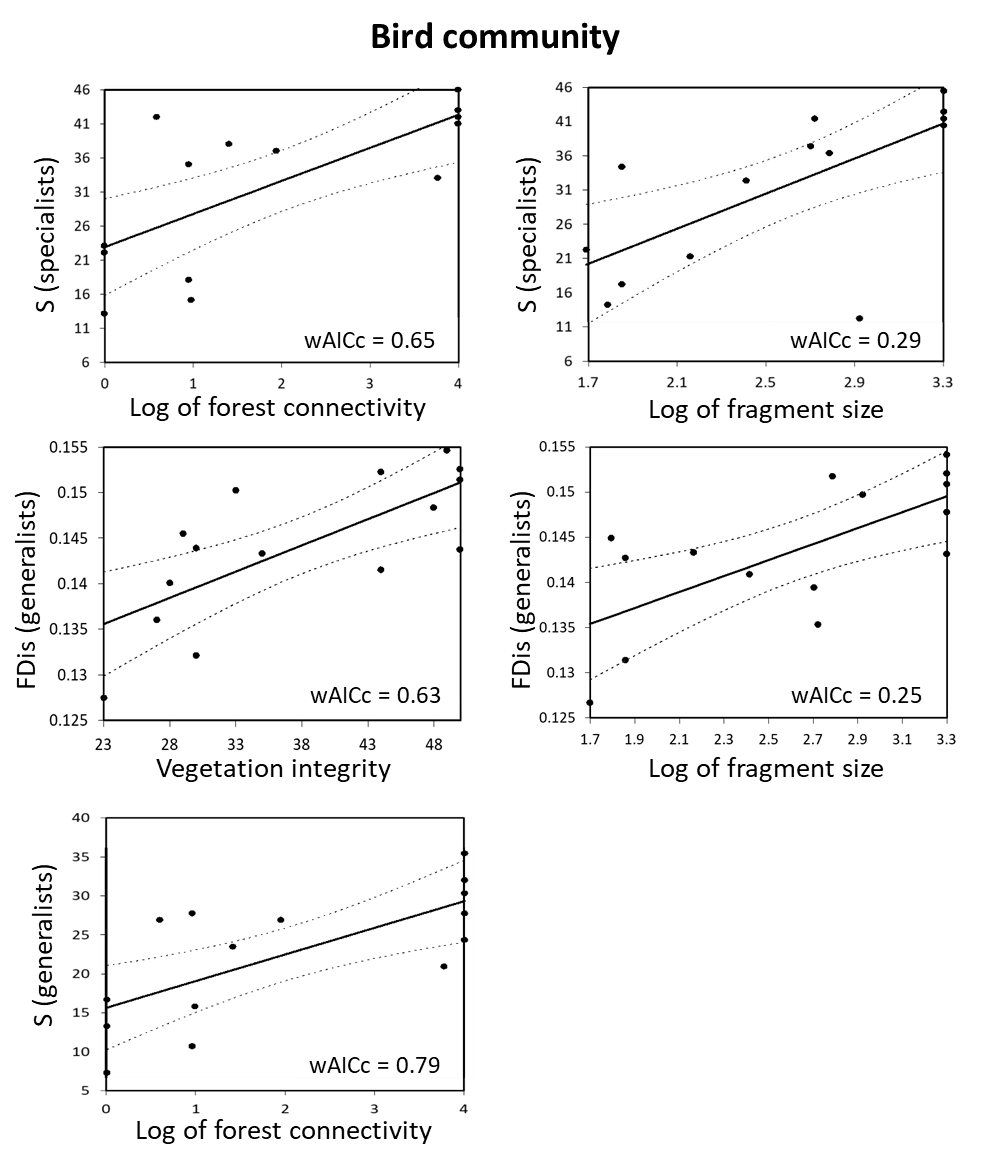


**Figure S2.** Best supported models of the relation between the explanatory variables and species richness (S) and functional diversity (FDis) of specialists and generalists species within passerines community in continuous and fragmented forest landscapes in southeast Brazil.


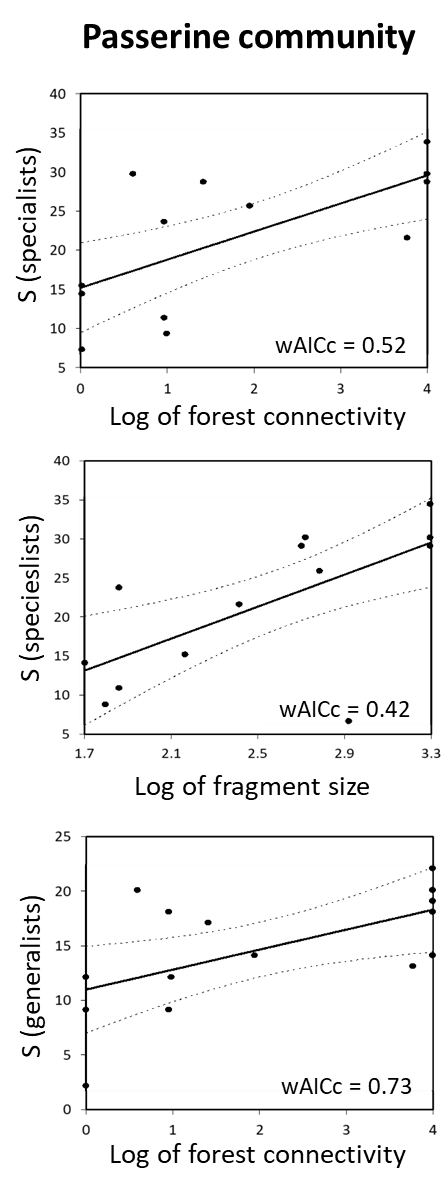


**References**

Baselga, A. (2010). Partitioning the turnover and nestedness components of beta diversity*. Global Ecology and Biogeography*, **19**(1), 134-143. https://doi.org/10.1111/j.1466-8238.2009.00490.x

Chesser, R. T. (2004). Molecular systematics of new world suboscines birds. *Molecular phylogenetics and evolution*, **32**(1), 11–24. https://doi.org/10.1016/j.ympev.2003.11.015

Fitzpatrick, J., Bates, J., Bostwick, K., Caballero, I., Clock, B., Farnsworth, A., Hosner, P., Joseph, L., Langham, G., Lebbin, D., Mobley, J., Robbins, M., Scholes, E., Tello, J., Walter, B., & Zimmer, K. (2004). Family Tyrannidae (Tyrant-flycatchers, Cotingas to pipits and wagtails). In: Hoyo, J., Elliott, A., & Christie, D. (Eds.), *Handbook of the birds of the world*. Barcelona: Lynx Edicions, pp. 170-462.

Gower, J. C. (1966). Some distance properties of latent root and vector methods used in multivariate analysis. *Biometrika*, **53**(3-4), 325-338. https://doi.org/10.1093/biomet/53.3-4.325

Krabbe, N. K., & Schulenberg, T. S. (2003a). Family Formicariidae (Ground-antbirds). In: Hoyo, J., Elliot, A., & Christie, A. (Eds), *Handbook of the Birds of the World*, Broabills to Tapaculos. Barcelona: Lynx Edicions, pp. 682–731.

Krabbe, N. K., & Schulenberg, T. S. (2003b). Family Rhinocryptidae (Tapaculos). In: Hoyo, J., Elliot, A., & Christie, A. (Eds), *Handbook of the Birds of the World*, Broabills to Tapaculos. Barcelona: Lynx Edicions, pp. 748-787.

Remsen, J. V. (2003). Family Furnariidae (Ovenbirds). In: Hoyo, J., Elliot, A., & Christie, A. (Eds.). *Handbook of the Birds of the World*. Barcelona: Lynx Edicions, pp. 162-357.

Snow, D. W. (2004a). Family Cotingidae (Cotingas, Cotingas to pipits and wagtails). In: Hoyo, J., Elliot, A., & Christie, A. (Eds). *Handbook of the Birds of the World*. Barcelona: Lynx Edicions, pp. 32-108.

Snow, D. W. (2004b). Family Pipridae (Manakins, Cotingas to pipits and wagtails). In: Hoyo, J., Elliot, A., & Christie, A. (Eds). *Handbook of the Birds of the World*. Barcelona: Lynx Edicions, pp.110-169.

Villéger, S., Mason, W., & Mouillot, D. (2008). New multidimensional functional diversity indices for a multifaceted framework in functional ecology. *Ecology*, **89**(8), 2290-2301. https://doi.org/10.1890/07-1206.1

Villéger, S., Grenouillet, G., & Brosse, S. (2013). Decomposing functional β‐diversity reveals that low functional β‐diversity is driven by low functional turnover in European fish assemblages. *Global Ecology and Biogeography*, **22**(6), 671-681. https://doi.org/10.1111/geb.12021

Whitney, B. M. (2003). Family Conopophagidae (Gnateaters) (Broabills to Tapaculos) In: Hoyo, J., Elliot, A., & Christie, A. (Eds). *Handbook of the Birds of the World*. Barcelona: Lynx Edicions, pp. 732-747.

Zimmer, K. J., & Isler, M. L. (2003). Family Thamnophilidae (Typical antbirds). In: Hoyo, J., Elliot, A., & Christie, A. (Eds.). *Handbook of the Birds of the World*. (Broabills to Tapaculos), Barcelona: Lynx Edicions, pp. 448-681.
